# Increased Constituent Ratios of *Klebsiella sp.*, *Acinetobacter sp.*, and *Streptococcus sp.* and a Decrease in Microflora Diversity May Be Indicators of Ventilator-Associated Pneumonia: A Prospective Study in the Respiratory Tracts of Neonates

**DOI:** 10.1371/journal.pone.0087504

**Published:** 2014-02-20

**Authors:** Wei Lu, Jialin Yu, Qing Ai, Dong Liu, Chao Song, Luquan Li

**Affiliations:** 1 Department of Neonatology, Children’s Hospital of Chongqing Medical University, Chongqing, China; 2 Pediatrics Research Institution, Children’s Hospital of Chongqing Medical University, Chongqing, China; 3 Ministry of Education Key Laboratory of Child Development and Disorder, Children’s Hospital of Chongqing Medical University, Chongqing, China; University of Tampere, Finland

## Abstract

Ventilator-associated pneumonia (VAP) is a common complication and cause of death in neonates on mechanical ventilation. However, it is difficult to define the causes of VAP. To understand the causes of VAP, we undertook a prospective study based on the diversity of the microflora in VAP. The experimental group consisted of newborns who suffered from respiratory distress syndrome (RDS) and VAP, while the control group suffered from RDS without VAP. Sputa were collected within 1, 3, and 5 days of ventilation and were divided into six groups. DNA was extracted from the samples, and the 16S rDNA was PCR amplified, separated using denaturing gradient gel electrophoresis (DGGE), cloned and sequenced. The resulting sequences were compared using BLAST. The DGGE pictures were measured, and the richness, Shannon-Wiener index, and cluster maps were analyzed. No differences were found regarding the constituent ratio of any genus between the Non-VAP and VAP group within 1 day after intubation. After 1 to 3 days, the constituent ratios of *Klebsiella sp.*, *Acinetobacter sp*., and *Streptococcus sp.* in the VAP group were higher than those in the Non-VAP group, and the ratios of *Serratia sp.* and *Achromobacter sp.* were lower. After 3 to 5 days, the ratios of *Klebsiella sp.*, *Acinetobacter sp., Serratia sp.,* and *Achromobacter sp.* were lower than those in the Non-VAP group. The richness and Shannon-Wiener index of the Non-VAP group were higher than those of the VAP group from 1 to 3 days after intubation, while no differences were found within 1 day and from 3 to 5 days. We conclude that during the first three days of intubation, the microflora diversity in the lower respiratory tract was reduced due to VAP, and the greater constituent ratios of *Klebsiella sp.*, *Acinetobacter sp.,* and *Streptococcus sp.* in the sputum may be indicators of VAP.

## Introduction

There is mounting evidence that shows that a variety of microorganisms exist in the human respiratory tract, gastrointestinal tract, skin, vagina, and other parts of the body. The number of microbial cells in the human body is 10 times greater than the number of human cells, and the number of microbial genes is 100 times greater than the number of human genes. We currently have identified only 5% of these symbiotic microorganisms. Nearly 95% of our symbiotic microorganisms are poorly understood, and we do not fully know the effects that these microorganisms have on our health, even though the Human Genome Project was completed in 2003 [1]. To this end, SCIENCE published a special issue entitled “Gut Microflora” in August 2012 that discussed the importance of studying the microflora. However, current research has focused primarily on the intestines, teeth, and vagina, while somewhat ignoring the microflora of the respiratory tract.

Ventilator-associated pneumonia (VAP) is a common complication and cause of death in newborns on mechanical ventilation [2]. However, it is difficult to define the exact causes of VAP [3]. Furthermore, due to the increased prevalence of drug-resistant bacteria in recent years and the limitations of culture and other detection methods, determining a course of treatment for VAP is quite difficult [4]. The complexity of the bacteria in the respiratory tract contributes to the diversity of this micro-ecosystem. Various pathogenic and commensal bacteria can collaborate, coexist, or restrict the growth of other bacteria, and commensal bacteria may become pathogenic under certain circumstances [5]. Therefore, to establish better clinical guidelines for the treatment of VAP, we need to understand the diversity and relationships between the microflora to detect pathogens.

Kellen found that more than 99% of the microorganisms in the environment are impossible or difficult to culture; therefore, traditional culture methods cannot fully reflect the entire composition of the microbial community [6]. Metagenomics (also known as microbial environmental genomics), as proposed by Schmidt, enables a comprehensive and objective understanding of the bacterial species and their interactions in the respiratory tract of pneumonia patients [7]–[8]. Sequence analysis based on the 16S rDNA is regarded as the gold standard for bacterial identification and classification [9]–[10]. 16S rDNA PCR-DGGE is one of the most commonly used methods for studying biological communities [11]. Until now, there have been no peer-reviewed papers regarding the bacterial diversity and dynamic changes in the neonatal respiratory tract during VAP. Therefore, a prospective study examining the composition and diversity of the microflora and their relationships with VAP was performed by collecting sputum specimens at different times. We sought to characterize the microflora during VAP and to improve the methods used to detect the pathogens responsible for VAP.

## Materials and Methods

### Ethics Statement

This study was approved by the Medical Ethics Committee of Chongqing Medical University, and written consent was obtained from the parents of the neonates in this study.

### Patient Selection

Newborns suffering from RDS in the NICU of the Children’s Hospital of Chongqing Medical University between December 2011 and June 2012 were invited to participate in this study. The experimental group contained newborns suffering from RDS combined with VAP (VAP group), and the control group contained infants that did not have VAP (Non-VAP group). The following exclusion criteria were used: 1) evidence of intrauterine infection in the RDS children; 2) RDS children that had been diagnosed with pneumonia, β-hemolytic *Streptococcus* infection, upper respiratory tract infection, sepsis, encephalitis, or other infectious diseases prior to intubation; and 3) mothers with a history of infections or who had used antibiotics during the last month of pregnancy.

The following diagnostic criteria were used for VAP: 1) the presence of rales or knock turbidity, as well as emerging purulent sputum, positive blood culture, or epidemic strains isolated by endotracheal suction; and 2) X-ray examination showing the presence of emerging pulmonary infiltrates, consolidation, cavities, or pleural effusions [3], [12]–[13].

### Sample Collection

The samples were collected within 1, 3, and 5 days of ventilation. For sputum sample collection, the suction tube of the sputum culture collector was placed deep into the collection tube, and 1–2 ml of sputum was aspirated using negative pressure. If the sputum was too thick to be collected, 1–2 ml of sterile saline was injected into the endotracheal tube, followed by five breathing cycles. Once the patient’s oxygen saturation recovered, the sputum was aspirated. All of the specimens were stored at −20°C immediately following collection [14].

### DNA Extraction

The sputum was centrifuged at 4°C and 10,000 rpm for 1 min, the supernatant was discarded, and the pellet was resuspended in 2 ml of sterile saline. The sample was mixed and centrifuged again following the above conditions. This wash was repeated twice, in accordance with the manufacturer’s instructions included with the Mini BEST Bacterial Genomic DNA Extraction Kit Ver2.0 (Takara). Filter-sterilized, double-distilled water was used as the negative control for the DNA extraction and PCR reactions [11].

### PCR Amplification

The following primers were synthesized by the Shanghai Biological Engineering Company and were designed according to the conserved V3 region of the bacterial 16S rDNA gene: 357f (5′-CGC CCG GGG CGC GCC CCG GGC GGGGCG GGG GCA GGG G CCTACG GGA GGC AGC AG-3′, including the 37 bp “GC” cap) and 518r (5′-ATT ACC GCG GCT GCT GG-3′). The amplifications were performed using an Eppendorf PCR machine (Eppendorf, Germany). The reaction volume was 50 µl and included 6 µl of template DNA, 25 µl of Premix Taq Version 2.0 (TaKaRa), 0.5 µl of each primer, and 18 µl of sterile ddH_2_O. The following reaction conditions were used: an initial denaturation at 94°C for 5 min; 10 cycles of denaturation at 94°C for 30 s, annealing at 61°C to 56°C (−0.5°C/cycle), and extension at 72°C for 1 min; 25 cycles of denaturation at 94°C for 30 s, annealing at 56°C for 30 s, and extension at 72°C for 1 min; and a final extension at 72°C for 7 min. Five microliters of the PCR products was resolved on a 2% agarose gel prepared with 1× TAE and 4S Green (Shanghai Biological Engineering Company). The target bands were approximately 195 bp in size. The remaining PCR products were stored at −20°C.

### DGGE

DGGE was performed using a Decode system (Bio-Rad). Twenty-five microliters of each PCR product was separated on an 8% polyacrylamide gel with a 35–65% linear gradient of urea and formamide by electrophoresis at 85 V and 60°C for 16 h. The gels were stained with SYBR Green I (100 Tektronix Biological Technology Company) and were imaged using a Herolab UVT-20 M/W ultraviolet transilluminator. All of the bands were excised, washed twice with 500 µl of sterile ddH2O, mashed, placed in 30 µl of nuclease-free water, and stored at 4°C overnight to elute the DNA. The supernatants were amplified with primers lacking the “GC” cap under the same reaction conditions described above. A 0.8% agarose gel was prepared with 1× TAE and 4S Green (Shanghai Biological Engineering Company). All of the amplification products were electrophoresed at 110 V for 20 min. The DNA in the target bands was recovered using an Agarose Gel DNA Purification Kit Version 2.0 (TaKaRa) and was stored at −20°C [14]–[15].

### Cloning and Sequencing

The PCR amplicons were cloned into a plasmid using the PMD18-T Vector system (TaKaRa), and the resulting clones were transformed into *Escherichia coli* DH5α competent cells (Tiangen, China). The cells were cultured overnight at 37°C on LB media containing ampicillin. One milliliter of this liquid was sent to Shanghai Biological Engineering Company for sequencing. The results were compared with the nucleotide databases in the NCBI GenBank using the BLAST tool.

### Bacterial Culture

The collected sputum oscillating fluid samples were inoculated onto Columbia blood agar plates and *Haemophilus influenzae* separated flat and were cultured for 18 to 48 h at 37°C. The resulting colonies were Gram stained and identified using a MicroScan WalkAway-40 automated bacterial identification and susceptibility test instrument.

### Analysis of Diversity and Similarity

Quantity One (BIO-RAD, America) software was used to measure the band number and the similarity in the DGGE images. The cluster maps were analyzed using the unweighted pair group method with arithmetic averages (UPGMA), and the Shannon-Wiener index (Shannon-Wiener diversity index) was calculated using the BIO-DAP software [14]–[16].

### Statistical Analysis

The SPSS17.0 statistical software was used to analyze the data. The data with a normal distribution were expressed as *x*±*s*. Comparisons between two groups were performed using the independent samples *t*-test, and comparisons between many groups were performed using one-way ANOVA or the pairwise LSD-*t* test when the variance was homogeneous. A non-parametric test was used when the variance was heterogeneous after calibration. The data without normal distributions are expressed as P50 (P25–P75) and were used in the non-parametric test method. Count data were used in the *χ* ^2^ test when n ≥40 and T ≥1, and the extract method was used when n <40 or T <1. *P*<0.05 indicates a significant difference. The pairwise comparisons of multiple count data were performed after the test level was fixed.

## Results

### Clinical Characteristics

There were 25 RDS infants that met the standards for inclusion in this study. Their treatments included immediate intubation, mechanical ventilation, and the use of pulmonary surfactants. At least one sputum sample from each patient was sent to the molecular clinic diagnosis center for culture in our hospital. Ten infants developed VAP within 1.8 to 2.9 days (P50 = 2.5) after intubation. Cefoxitin was empirically used in the two groups. The members of the VAP group with positive culture results after diagnosis were treated with piperacillin/tazobactam, cefoperazone/sulbactam, etc., according to the susceptibility data. The clinical data are shown in Table 1. There were no significant differences between the total intubation times of the two groups. A total of 32 sputum samples were sent for culture, and the detection ratio was 40.6%.

**Table 1:** Clinical characteristics of the 25 infants included in this study.

	Non-VAP (n = 15)	VAP (n = 10)	Statistics	*P*
Gender (Male)	10	7	χ^2^ = 0.006	0.939
Gestational age (*x*±*s*, week)	30.4±2.3	30.0±1.9	*t* = 0.353	0.556
Birth weight (*x*±*s*, g)	1735±156	1695±167	*t* = 1.024	0.318
Total intubation duration [P50 (P25–P75), day]	4.1 (1.5–5.8)	10.3 (4.3–13.7)	–	0.000^a^
Sputum culture results			–	0.715^b^
Negative (n)	8	4	–	–
*Klebsiella pneumoniae subspecies* (n)	5	4	–	–
*Escherichia coli* (n)	0	1	–	–
*Acinetobacter baumannii* (n)	2	1	–	–
Antibiotic	Cefoxitin	Piperacillin/tazobactam, Cefoperazone/sulbactam, Imipenem, etc.	–	–
Prognosis			–	0.653^b^
Improved (n)	9	7	–	–
Healed (n)	5	2	–	–
Died (n)	1	1	–	–

a Non-parametric test; b: Fisher’s exact test.

### Sample Collection, DNA Extraction, and PCR

Fifty-two sputum samples were collected. Target bands approximately 195 bp in size were obtained from 43 samples (82.7%) after extraction, amplification, and analysis by 2% agarose gel electrophoresis. These data are shown in Table 2. The negative control group did not generate the target band.

**Table 2:** Groupings based on the DNA amplification of 43 samples.

Time	Non-VAP	VAP
D1	10	8
D3	7	7
D5	5	6
Total	22	21

D1∶0–1 day after intubation; D3: −3 days after intubation; D5: −5 days after intubation.

### DGGE Pictures

**Figure 1:**
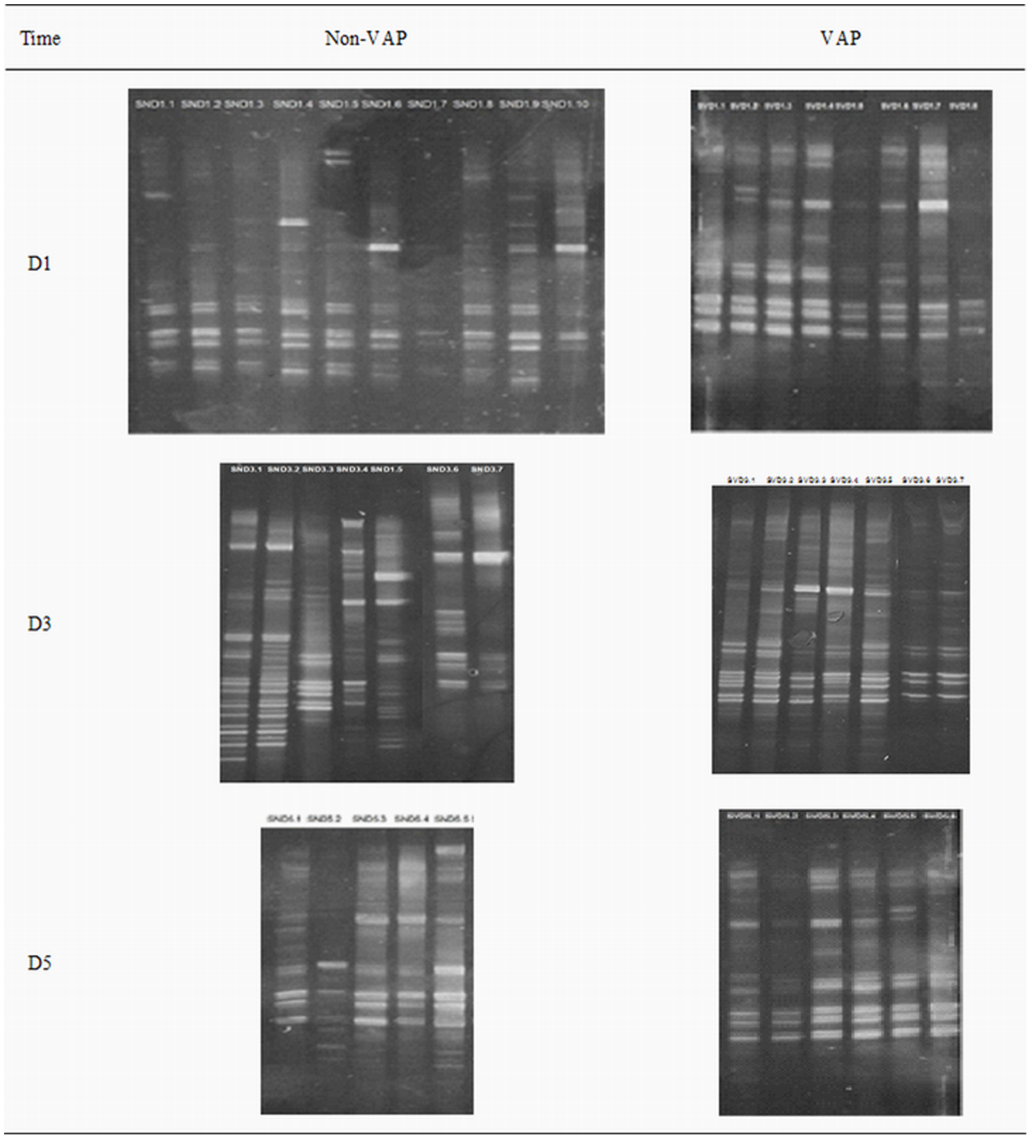
DGGE pictures of the DNA amplified products.

All of the 43 samples mentioned above had visible bands after DGGE, and these images are shown in Figure 1. The presence of more than one band in a lane indicates that multiple bacterial genera were present in the sample [17]. The varying thicknesses of the bands are due to different optical densities. Thicker and darker bands indicate a greater optical density and higher DNA content. The positions of the bands also differ. There were many common bands and a few specific bands, indicating that certain bacteria were found in all of the samples in addition to some sample-specific bacterial species.

### Sequencing Results

Nine species were detected after the DGGE bands were isolated, cloned, and sequenced (Table 3.).

**Table 3:** Sequencing results of the DGGE bands.

NCBI BLAST result	Accession number	Identity (%)
*Serratia sp.*	KC182731.1	100
*Achromobacter sp.*	HE613447.1	100
*Klebsiella sp.*	KC354804.1	100
*Staphylococcus sp.*	JX849039.1	100
*Acinetobacter sp.*	KC245151.1	99
*Streptococcus sp.*	JX861486.1	100
*Macrococcus sp.*	HQ238716.1	100
*Brevundimonas sp.*	JX950099.1	99
*Actinomyces sp.*	HM854563.1	99

### Composition of the Microflora

**Figure 2:**
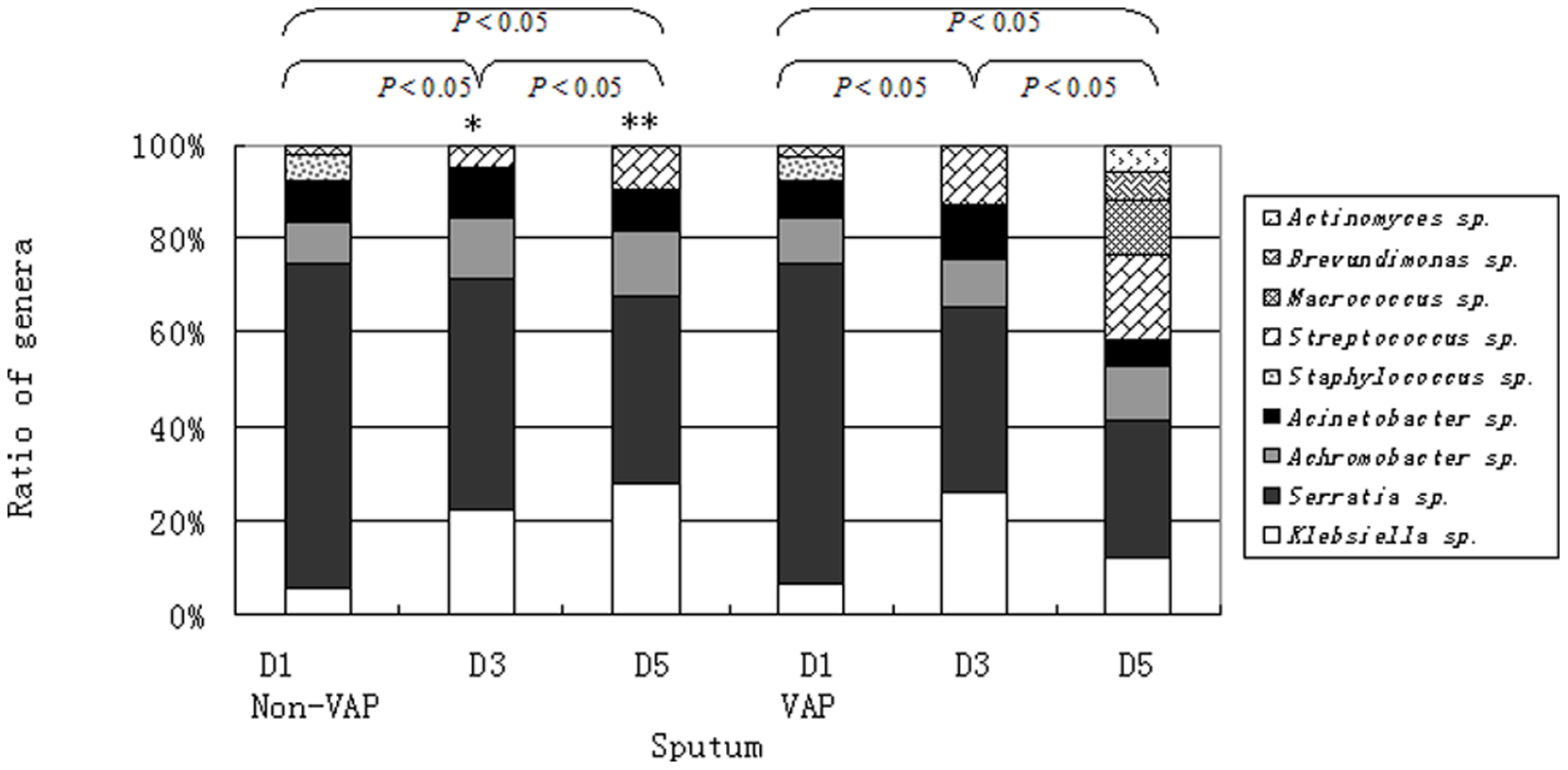
Composition ratio of the genera in each group. The difference in the composition ratio of the genera between the Non-VAP D3 group and the VAP D3 group was significant; *, *P*<0.05. The difference in the composition ratio of the genera between the Non-VAP D5 group and the VAP D5 group was significant; **, *P*<0.05.

The variety of the genera in each group and their composition ratios are shown in Figure 2. Nine genera were detected in the lower respiratory tract. There were 5 basic genera detected in the 6 groups, including *Klebsiella sp.*, *Serratia sp.*, *Achromobacter sp.*, *Streptococcus sp.*, and *Acinetobacter sp. Staphylococcus sp.* only appeared 1 day after intubation. No differences were found regarding the constituent ratio of any genus between the Non-VAP and VAP groups within 1 day after intubation. From 1 to 3 days of intubation, the constituent ratios of *Klebsiella sp.*, *Acinetobacter sp*., and *Streptococcus sp.* in the VAP group were higher than those in the Non-VAP group, and the ratios of *Serratia sp.* and *Achromobacter sp.* were lower. From 3 to 5 days, the constituent ratio of *Streptococcus sp.* was higher in the VAP group than in the Non-VAP group, and the ratios of *Klebsiella sp.*, *Acinetobacter sp., Serratia sp.*, and *Achromobacter sp.* were lower.

### Comparison of the Detection Rate between the Two Methods

In this study, the detection rate by sequencing was 82.7% (43/52). However, the detection rate for the culture method was 40.6% (χ^2^ = 3.411, *P* = 0.047). This result demonstrates that the 16S rDNA PCR-DGGE method combined with cloning and sequencing was a more effective method for the detection of bacteria than the culturing method.

### Analysis of Diversity

**Figure 3:**
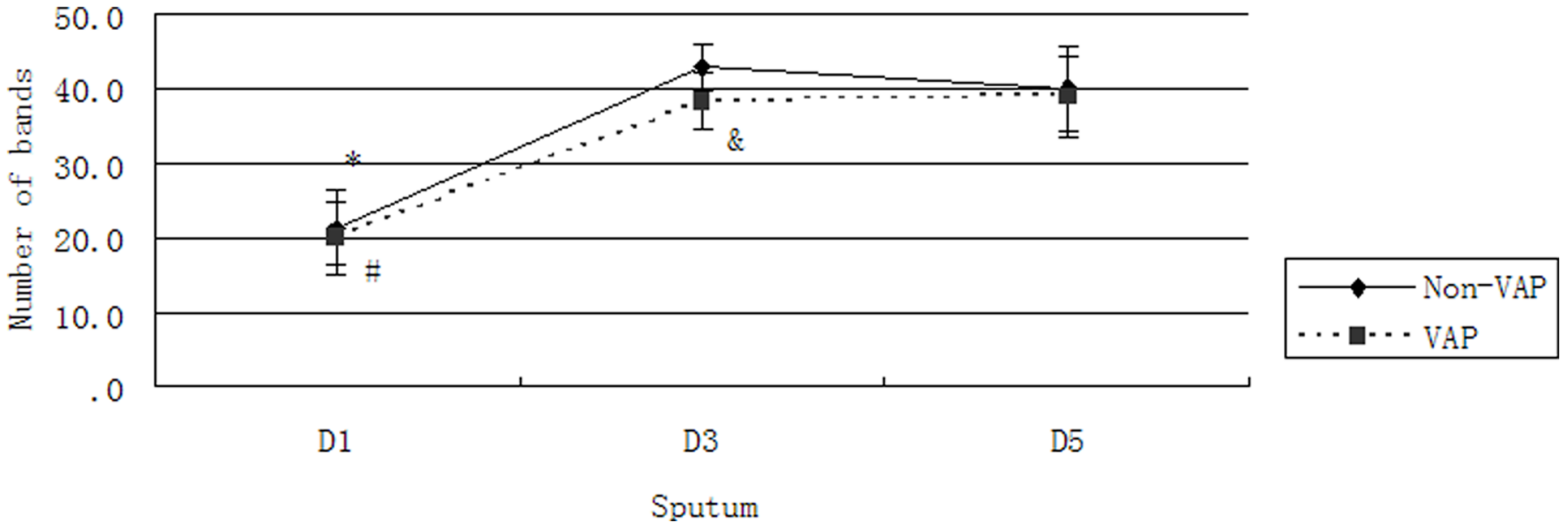
Number of bands in each group. The number of bands in the Non-VAP D1 group was lower than that in the Non-VAP D3 and D5 groups; *, *P*<0.05. The number of bands in the VAP D1 group was lower than that in the VAP D3 and D5 groups; #, *P*<0.05. The number of bands in the Non-VAP D3 group was greater than that in the VAP D3 group; &, *P*<0.05.

An increased number of bands within a lane indicated the increased bacterial diversity of that sample [17]. The number of bands in each group is shown in Figure 3. The number of bands in the VAP and Non-VAP groups initially increased, and this increase was then maintained, indicating that the richness of the Non-VAP group was higher than that of the VAP group between 1 and 3 days after intubation; however, no differences were observed on the first day or between 3 and 5 days.

**Figure 4:**
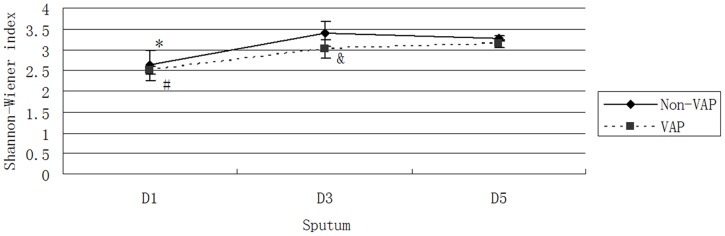
Shannon-Wiener index for each group. The Shannon-Wiener index for the Non-VAP D1 group was lower than that for the Non-VAP D3 and D5 groups; *, *P*<0.05. The Shannon-Wiener index for the VAP D1 group was lower than that for the VAP D3 and D5 groups; #, *P*<0.05. The Shannon-Wiener index for the Non-VAP D3 group was higher than that for the VAP D3 group; &, *P*<0.05.

The Shannon-Wiener index comprehensively reflected the changes in the genera and quantities of bacteria. An increase in the number of genera and quantities of each bacterium indicated the increased bacterial diversity of the sample [17]. In this experiment, the number of bacteria was indirectly reflected by the optical density value of the bands. The Shannon-Wiener indices of each group are shown in Figure 4. These values demonstrated that the overall trends and comparative relations were consistent with richness.

### Similarity and Cluster Analysis of Microflora

**Figure 5:**
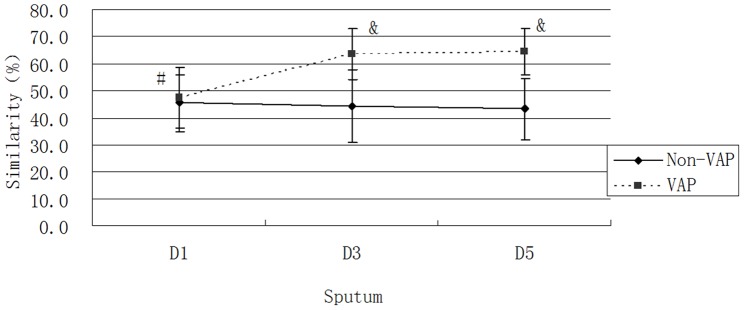
Similarity in each group. The similarity in the VAP D1 group was lower than that in the VAP D3 and D5 groups; #, *P*<0.05. The similarities in the VAP D3 and D5 groups were higher than that in the Non-VAP D3 and D5 groups; &, *P*<0.05.

**Figure 6:**
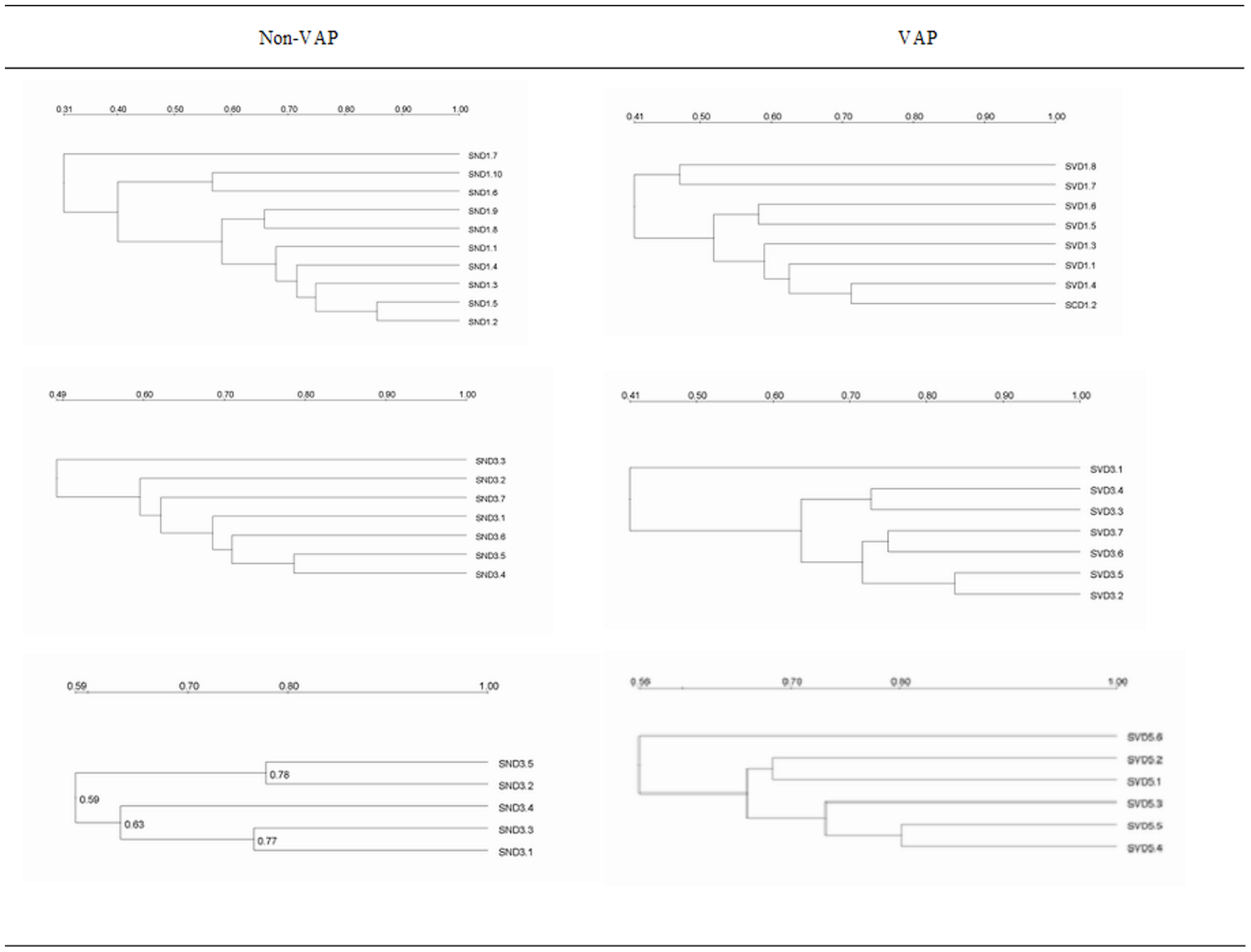
Cluster maps of each group.

The similarity results for each group are shown in Figure 5, and the cluster analysis is shown in Figure 6. The similarity of the Non-VAP group was always lower than that of the VAP group after 1 day of intubation, which indicated that the microflora composition of each sample in the Non-VAP group tended to be more different and thus demonstrated greater variation within the group [16].

## Discussion

Nine genera were detected in the lower respiratory tract. The number, type, and composition of the bacteria present changed dramatically during the course of intubation. Five common genera were shared across the samples. The trends for *Streptococcus sp.* in the Non-VAP group and the VAP group were similar. Reports have shown that streptococci can generate inducible factor AI-2 to moderate biofilm formation by other bacteria. However, the potential role of the streptococci in promoting the growth of pathogenic bacteria and its involvement in the formation of VAP should be studied further [18]–[19]. We found that the following six genera were present in the sputum within one day of intubation: *Klebsiella sp.*, *Serratia sp.*, *Achromobacter sp.*, *Acinetobacter sp.*, *Staphylococcus sp*., and *Streptococcus sp.* Their proportions were the same in the Non-VAP group and the VAP group. During one to three days after intubation, there were only five genera present in the sputum. *Staphylococcus sp*. may be suppressed by *Klebsiella sp., Acinetobacter sp.*, and *Streptococcus sp.* due to the observed increases in the composition ratios of these organisms. Although the Non-VAP patients did not have complicated infections, they routinely received cefoxitin, so this genus was suppressed. Although the number and type of genera were the same in the Non-VAP and VAP groups when D3 was compared to D1, their overall composition ratios were different. The constituent ratios of *Klebsiella sp.*, *Acinetobacter sp.*, and *Streptococcus sp.* in the VAP group were greater than those in the Non-VAP group. *Klebsiella* subspecies and *Acinetobacter baumannii* were cultured in this study, and the constituent ratios of *Klebsiella sp.* and *Acinetobacter sp.* were larger in the VAP group than in the Non-VAP group. In other words, if a certain type of cultivated bacteria belongs to a genus whose composition ratio was higher than that of the control group, RDS complicated with VAP should be considered, and these cultivated bacteria are most likely pathogenic. Thus, determining the composition ratio of a species may provide additional evidence to help identify the pathogenic bacteria [14]. The constituent ratios of *Klebsiella sp.*, *Acinetobacter sp.*, and *Serratia sp.* in the VAP group and the Non-VAP group after 3 to 5 days of intubation were the opposite of the results obtained after 1 to 3 days. The susceptibility testing results showed that the *Klebsiella* subspecies and *Acinetobacter baumannii* were resistant to cefoxitin; therefore, these two bacteria may continue to grow in the Non-VAP group patients who received cefoxitin, while their growth was suppressed in the VAP group that was treated with different antibiotics [20]–[22]. These differences could result in the higher constituent ratios of *Klebsiella* subspecies and *Acinetobacter baumannii* in the Non-VAP group.

It would be more comprehensive to combine the richness measurements and the Shannon-Wiener index. In general, their trends were similar. In this experiment, the richness and Shannon-Wiener index of the VAP group were lower than those of the Non-VAP group during 1 to 3 days after intubation. This result indicates that the diversity of the microflora in the VAP group was lower than that in the Non-VAP group. However, the diversity indices of the VAP group and the Non-VAP group were not different 3 to 5 days after intubation. There are four possible reasons for this observation: most of the VAP children may have been improving, the amounts of drug-resistant bacteria were increasing in the VAP patients, the bacteria in the Non-VAP group were inhibited by the antibiotics, or the Non-VAP group along with the tendencies to VAP. The above four reasons concur with our observation that the diversity and quantities of bacteria decreased in the Non-VAP group but increased in the VAP group. Therefore, the richness and Shannon-Wiener index were the same for the two groups. However, the specific mechanism by which this occurs should be studied further, and every case in the clinic needs to be carefully analyzed. Meanwhile, the number of samples in this experiment was relatively small and should be expanded to improve the reliability of VAP prediction.

Additionally, the similarity in the VAP group was higher than that in the Non-VAP group after one day of intubation. This result indirectly indicates that the diversity of the VAP group was restricted [17] and may be valuable in predicting VAP.

In this study, nine bacterial genera were detected by 16S rDNA sequencing, and three genera were detected by culturing sputum samples. *Klebsiella pneumoniae* subspecies and *Acinetobacter baumannii* were identified using both methods. The three cultured bacteria are all common opportunistic pathogens [23]–[24], but *Klebsiella pneumoniae* subspecies and *Acinetobacter baumannii* also appeared in 7 Non-VAP patients. Furthermore, the constituent ratios of the pathogens were the same in the Non-VAP group and the VAP group, indicating that we cannot diagnosis VAP based on the culture results alone. This study also showed these two types of bacteria colonized the lower respiratory tracts of RDS patients. Therefore, we should comprehensively consider clinical manifestations, chest X-rays, and other laboratory parameters when diagnosing VAP [25]–[26].

The two detection methods also greatly differed in that no pathogens were cultured from four VAP patients, and seven species, *Serratia sp., Achromobacter sp., Streptococcus sp., Staphylococcus sp., Actinomyces sp., Brevundimonas sp.,* and *Macrococcus sp.*, that were detected by sequencing were not cultured. These observations show that the results of the 16S rDNA sequencing are more comprehensive and that this method can be used to detect bacteria that can be cultured, as well as those that cannot be cultured. Certain bacteria, such as *Streptococcus sp*., grow poorly in normal medium. Certain bacteria belong to the normal microflora. The effects of the collection times and antibiotic treatment can result in negative cultures. For example, *Staphylococcus sp.* was only observed at one day after intubation, while *Actinomyces sp., Brevundimonas sp.*, and *Macrococcus sp.* were found at 3 to 5 days after intubation [4], [27]–[29]. Therefore, 16S rDNA sequencing may be considered an important supplement to the traditional culture method because of its higher detection efficiency.

There are still many limitations of this study, including we could not use qPCR as a supplement to DGGE because there were many difficulties in standardizing the initial quantitation of the sputum, the small sample size, the fact that the sputum samples from normal newborns cannot be collected from a normal control group, the lack of a control group that was not treated with antibiotics, the fact that the positive rate of bacterial DNA extraction did not reach 100%, the fact that we could only designate the sequences of genera that had deposited in the Genebank, and inaccurate sequencing results at the species level. In the future, the time of specimen collection should be extended, a matched control group should be established, patients who improved and patients who died should be grouped separately, and species should be identified using universal primers that anneal to multiple conserved regions or using species-specific primers. Further research should implement the above improvements and focus on the variations in the bacterial community structures and diversity in children with RDS and VAP.

In conclusion, within one day after intubation, there were no differences in the microflora structure, diversity, or similarity between the Non-VAP group and the VAP group. During one to three days of intubation, the microflora in the lower respiratory tract became less diverse in the VAP group. The higher constituent ratios of *Klebsiella sp., Acinetobacter sp.*, and *Streptococcus sp.* in the sputum may be indicators of VAP. After three to five days of intubation, the situation becomes more complicated.
