# Correction: Increased Constituent Ratios of *Klebsiella sp.*, *Acinetobacter sp.*, and *Streptococcus sp.* and a Decrease in Microflora Diversity May Be Indicators of Ventilator-Associated Pneumonia: A Prospective Study in the Respiratory Tracts of Neonates

**DOI:** 10.1371/journal.pone.0105928

**Published:** 2014-08-12

**Authors:** The PLOS ONE Staff

There are some missing grant numbers in the funding statement for this article. Please refer to the correct funding statement below:

This work was supported by grant No. 8107513, No. 81370744, and No. 30901279 from National Natural Science Foundation of China (General Program). The funders had no role in study design, data collection and analysis, decision to publish, or preparation of the manuscript.
